# Supplementary material for: Patterns of Intron Gain and Loss in Fungi
Source: PLoS Biol. 2004 Nov 30;2(12):e422. doi: 10.1371/journal.pbio.0020422 (PMC532390; doi:10.1371/journal.pbio.0020422)
Supplement: Table S1 — Also available at http://genes.mit.edu/NielsenEtAl/. (4.3 MB ZIP). [file pbio.0020422.st001.zip › NielsenEtAl/html/1309.html]

AN2734.1.NCU07408.1.MG04467.1.FG06827.1


```
 CLUSTAL W (1.82) Multiple Sequence Alignments - Introns Inserted


Sequence 1: NCU07408.1	313 aa
Sequence 2: FG06827.1	311 aa
Sequence 3: MG04467.1	313 aa
Sequence 4: AN2734.1	312 aa
Alignment Length: 314 aa
Number Identitical Residues: 211 aa
Alignment Score (without introns) 8881


MG04467.1 	MGGKTGNKAGYFDKLKGLLEEYKSIFIVGVDNVSSQQMHEIRQSLRGEAVVLMGKNTM0V
NCU07408.1	MGGKSANKAGYFDKLKGLLEDYRSIFIVSVDNVSSQQMHEIRQSLRGEAVVLMGKNTM0V
FG06827.1 	MGAK-SNKSLYFDKLKGLLEKYTSIFIVEIDNVSSQQMHEIRHALRNKGVVLMGKNTM0V
AN2734.1  	MGGKSATKAAYFEKLKNLLDEYKTVFIVGVDNVSSQQMHEIRIALRGEGVVLMGKNTM0V
          	**.*:..*: **:***.**:.* ::*** :************ :**.:.********* *

MG04467.1 	RRALKTFVSESPEYERLLPYVKG~NVGFVFTNSDLKEIRDKILQNRVRAPARAGAVAPDD
NCU07408.1	RRALKTFVADTPEYERLLPFVKG~NVGFVFTNGDLKEVRDKILANKVAAPARAGAIAPAD
FG06827.1 	RRALKTFVADSPEYERLLPHVKG~NVGFVFTNEDLKEVRDVILANKVAAPARAGALAPAD
AN2734.1  	RRALKGFINDNPEYERLLPHVKG1NVGFIFTNGDLKETKDKILSNRVAAPARAGAVAPAD
          	***** *: :.********.*** ****:*** **** :* ** *:* *******:** *

MG04467.1 	VWIPAGNTGMEPGKTSFFQALGVPTKIARGTIEITTDLKLVEAGGKVGASEATLLNMLNI
NCU07408.1	VWIPAGNTGMEPGKTSFFQALGVPTKIARGTIEITTDLKLVEAGAKVGPSEATLLNMLNI
FG06827.1 	VWVPAGNTGMEPGKTSFFQALGVPTKIARGTIEITTDLKLVEAGSKVGPSEATLLNMLNI
AN2734.1  	VWIPAGNTGMEPGKTSFFQALGVPTKIARGTIEITTDLKLVEEGNKVGPSEATLLNMLNI
          	**:*************************************** * ***.***********

MG04467.1 	SPFTYGLTVEQVYDQGNCFPSSVLDIGEEQLLKSFSSAIATIAAISLALDFPTWPSVIHS
NCU07408.1	SPFTYGMGIAQVYDQGNTFPSDVLDISEEQLLKAFSTAITTIASLSLALNFPTLPSVIHS
FG06827.1 	SPFTYGMGISQVYDQGQTFPPSVLDVGEEQLLKTLSQAIATIATISLALNFPTLPSVLHS
AN2734.1  	SPFTYGMTIQQVYQDGQTFSAAVLDIEEEQLLKVFSSAINTITSISLAVNYPTLPSVIHS
          	******: : ***::*: *.. ***: ****** :* ** **:::***:::** ***:**

MG04467.1 	VVNSYKNILSIALETEYSFPAVEELKDRIANPDAYASAAPAAGGAGGGDAPAAEAKKDES
NCU07408.1	VVNAYKKVLAVAIETEISWPEIEELKDRIANPEAYASAAPAASTEAAPAAGKAEEKEEEK
FG06827.1 	LVNSYKKVLAVAVVTEVSWPEIEQLKDRIANPDAYASAAPAAAASGGDAPAAEEAKKDES
AN2734.1  	VVNSYKKVLAVAVETEYSWPEIEELKDRIANPEAYASAAPAAAAGGAAPAAAAEAAPAEE
          	:**:**::*::*: ** *:* :*:********:*********.  ..  .   *    *.

MG04467.1 	EEEQSDEDMGFG-LF~D
NCU07408.1	EES-DEDDGGFGGLF2D
FG06827.1 	EE--EEDDEGFGGLF2D
AN2734.1  	AEE-SDEDMGFG-LF~D
          	 *. .::* *** ** *
```
